# Elevated CO_2_ Atmosphere Minimizes the Effect of Drought on the Cerrado Species *Chrysolaena obovata*

**DOI:** 10.3389/fpls.2016.00810

**Published:** 2016-06-14

**Authors:** Vanessa F. Oliveira, Emerson A. Silva, Maria A. M. Carvalho

**Affiliations:** ^1^Núcleo de Pesquisa em Fisiologia e Bioquímica, Instituto de Botânica, São PauloBrazil; ^2^Universidade de Mogi das Cruzes, São PauloBrazil; ^3^Programa de Pós-Graduação em Biodiversidade Vegetal e Meio Ambiente, Instituto de Botânica, São PauloBrazil

**Keywords:** Cerrado, climate change, fructan active enzymes, high CO_2_, inulin, reserve organs, water deficit

## Abstract

*Chrysolaena obovata* stores inulin in the rhizophores, associated with drought tolerance. While crop plants are widely studied concerning the interactive effects of high [CO_2_] and drought, few studies reported these effects in native species. Here, we evaluated the combined effects of these factors on water status and fructan metabolism in *C. obovata*, a native Cerrado species. Two lots of plants were kept at 380 and 760 ppm CO_2_ in open-top chambers. In each, [CO_2_] plants were divided into four groups and cultivated under different water availability: irrigation with 100 (control), 75 (low), 50 (medium), and 25% (severe drought) of the water evapotranspirated in the last 48 h. In each, water treatment plants were collected at 0, 9, 18, and 27 days. On day 27, all plants were re-watered to field capacity and, after 5 days, a new sampling was made. Water restriction caused a decrease in plant moisture, photosynthesis, and in enzymes of fructan metabolism. These changes were generally more pronounced in 25% plants under ambient [CO_2_]. In the later, increases in the proportion of hexoses and consequent modification of the fructan chain sizes were more marked than under high [CO_2_]. The results indicate that under elevated [CO_2_], the negative effects of water restriction on physiological processes were minimized, including the maintenance of rhizophore water potential, increase in water use efficiency, maintenance of photosynthesis and fructan reserves for a longer period, conditions that shall favor the conservation of this species in the predicted climate change scenarios.

## Introduction

For much of the earth’s history, the concentration of carbon dioxide (CO_2_) in the atmosphere has been about 285 μmol mol^-1^ (ppm; Reddy et al., 2010). However, since the industrial revolution, that number has climbed steadily until February 2015, reaching an average of 400.2 μmol mol^-1^ in Mauna Loa, Hawaii (USA; Earth System Research Laboratory [ESRL], 2015). It has been predicted that the increasing atmospheric CO_2_ may cause global warming as well as changes in precipitation patterns (Kimball et al., 2001; Solomon et al., 2009). Under these conditions, drought episodes are predicted to be more frequent, severe and erratic and will possibly affect regions not currently subjected to drought (Allen, 1994; Centritto et al., 1999; Wiltshire et al., 2013). Climatic models suggest that in South America there will be an increase in precipitation; however, this phenomenon should be limited to a few months of the year, leaving long periods of drought (IPCC, 2013). In Brazil, for example, the Amazon could show a decrease in water availability (Wiltshire et al., 2013), which could lead to replacement of the Amazon Forest by Cerrado (IPCC, 2013).

The Cerrado, a tropical savanna in the central region of Brazil, presents an average precipitation of 800–2000 mm, with a distinct dry season during winter (3–6 months) and a wet summer (Dias, 1992). High irradiances, elevated air temperatures and low relative humidity impose a high evaporative demand during the dry season, when evapotranspiration greatly exceeds rainfall, depleting the upper soil layers of water (Franco, 1998, 2002; Quesada et al., 2008). The Cerrado vegetation presents seasonal growth and a series of adaptive strategies to overcome adverse environmental conditions of this biome. One strategy of herbaceous plants is the presence of thickened underground organs that accumulate photoassimilates (Mantovani and Martins, 1988). For example, in a restricted Cerrado area of the Reserva Biológica e Estação Experimental de Mogi Guaçu, São Paulo, Brazil, there is a high number of Asteraceae species that accumulate fructans as reserve compounds in the underground organs. These carbohydrates are synthesized when assimilation exceeds the demand for energy, mainly during the summer months, and mobilized for aerial growth or following a stress episode (Carvalho et al., 2007; Rigui et al., 2015).

Fructans are regarded as second to starch in importance as storage carbohydrates, being present in approximately 15% of the contemporary Angiosperm flora (Hendry and Wallace, 1993). Species containing fructans are distributed within a diverse range of families including Poaceae and Asteraceae. As a reserve compound, fructans are accumulated and mobilized throughout the phenological cycle of the plants. The occurrence of fructan metabolism in plants has been associated with cold and drought tolerance (e.g., Pontis, 1989; Tognetti et al., 1990; De Roover et al., 2000; Garcia et al., 2011). Accumulation of low DP (degree of polymerization) fructans and sucrose either by synthesis or by breakdown of higher DP molecules is commonly observed under these stresses. Since mild cold and drought limit growth to a greater extent than photosynthesis, accumulation of sucrose occurs and favors the activation of sucrose: sucrose 1-fructosyltransferase (1-SST, EC 2.4.1.99) and fructan: fructan 1-fructosyltransferase (1-FFT, EC 2.4.1.100; Hare et al., 1998; De Roover et al., 2000). These activities provide the driving forces for fructan synthesis and prevent the retro inhibition of photosynthesis, constituting an advantage for plants that accumulate fructan. Fructan hydrolysis is catalyzed by the action of fructan 1-exohydrolase (1-FEH, EC 3.2.1.153) whose activity is increased by plant defoliation, cold, and drought, leading to accumulation of low DP fructans and sucrose (Portes et al., 2008; Asega et al., 2011; Garcia et al., 2011, 2015).

Fructan metabolism is regulated by the presence of sucrose which is dependent on photosynthesis and radiation use efficiency (Edelman and Jefford, 1968; Incoll and Neales, 1970; Wiemken et al., 1986). Nevertheless, studies showing changes in photosynthesis and its effects on fructan metabolism under different environmental conditions are scarce. Oliveira et al. (2010) reported increased photosynthesis in plants of *Chrysolaena obovata* (Less.) Dematt. under high CO_2_, being the extra carbon mainly converted into rhizophore biomass and increased fructan yield. Study conducted with this same species showed that under severe drought, loss of aerial organs and arrest of photosynthesis occurred without changes in fructan levels (unpublished). On the other hand, Vandoorne et al. (2012) reported that fructan levels and net assimilation rates were maintained in plants of *Cichorium intybus* L. under drought while growth and fructan production were reduced, indicating that photosynthesis in this species is resilient to water stress.

Studies conducted with fructan-accumulating species of the Cerrado revealed their excellent potential for understanding the effects of environmental stresses on growth, biomass allocation and fructan metabolism (Carvalho et al., 2007). One of these species is the Asteraceae *C. obovata*, previously named *Vernonia herbacea* (Vell.) Rusby that accumulates inulin-type fructans in the underground reserve organs, the rhizophores (Carvalho and Dietrich, 1993).

The rise in atmospheric CO_2_ concentration is not an isolated factor and studies on the interactions between this and water stress are still limited to predict their impact on plant growth and productivity (Luo and Mooney, 1999). In addition, it is well known that the increase in biomass (Rogers et al., 1984; Curtis and Wang, 1998), and the changes in photosynthesis and water relations (Gunderson and Wullschleger, 1994; Saxe et al., 1998) caused by elevated CO_2_ are dependent on the availability of other potentially limiting resources, such as nutrients (Long, 1991; Chaves and Pereira, 1992; Field et al., 1992; Stitt and Krapp, 1999).

Previous studies showed that higher CO_2_ concentration promoted increases in height (40%), photosynthesis (63%) and biomass of aerial (32%) and underground (47%) organs of *C. obovata* (Oliveira et al., 2010). Although fructan concentration based in mass unit remained unchanged in the rhizophores, total fructan yield was higher in plants maintained under elevated [CO_2_], due to increased rhizophore biomass. An increase in water use efficiency (WUE, 177%) was also observed in these plants.

With the exception of a few studies (e.g., Coleman et al., 1991; Bassow et al., 1994; Roden and Ball, 1996; Duan et al., 2014), elevated CO_2_ has been shown to mitigate stress impact. For example, in *Pinus taeda* L. and *Quercus rubra* L., elevated CO_2_ reduced the impact of extreme heat and low soil water availability on photosynthesis parameters (Ameye et al., 2012; Bauweraerts et al., 2013). Similarly, the above-ground biomass production in grassland ecosystems in response to multiple stressors was reduced to a lesser extent under elevated CO_2_ than in current atmospheric CO_2_ concentration due to changes in antioxidant defenses and in carbohydrate metabolism (Naudts et al., 2014). High [CO_2_] also reduced the negative effects of drought on sorghum grain quality by delaying physiological and metabolic responses to this stress (De Souza et al., 2015). Plants of *Viguiera discolor* Baker under water restriction maintained tissue turgor and high CO_2_ assimilation rates when submitted to high atmospheric CO_2_ concentration (Oliveira et al., 2013). Under these conditions, plants also presented a delay in fructan mobilization. These physiological responses in *V. discolor*, together with the maintenance of high fructan levels for longer periods in plants under water deficit, suggested that high [CO_2_] minimized the negative effects of drought and contributed to their long term survival.

*Viguiera discolor* and *C. obovata* co-occur in the herbaceous layer of the Brazilian Cerrado, with a high number of inulin-accumulating species of Asteraceae; however, there seems to be no competition between them. Both species have been extensively studied (Carvalho et al., 2007) and *V. discolor* contains unusually long fructan chains, with an estimated maximum DP of 170 (Isejima and Figueiredo-Ribeiro, 1993), while the maximum fructan chain size in *C. obovata* is DP 64 (Portes and Carvalho, 2006). Although both species present many similarities such as high fructan content in reserve organs, and both are part of the same biodiverse-rich functional group in the Cerrado (Franco et al., 2014), their responses to high CO_2_ and drought may not be the same, since these responses are in many cases, species-specific. Therefore, in this study we proposed to evaluate the combined effects of high CO_2_ and drought on physiological responses and fructan metabolism in plants of *C. obovata*, aiming to contribute to the understanding of the behavior of an important functional group of the Cerrado vegetation in the light of future climate change scenarios.

## Materials and Methods

### Plant Material and Gowth Conditions

Plants of *Chrysolaena obovata* (Less.) Dematt. were obtained through vegetative propagation from rhizophore fragments, as described in Carvalho et al. (1998), using adult plants collected in a preserved Cerrado area in Mogi Guaçu, SP, Brazil, (22°18′S, 47°11′W). After 2 months they were transferred to 3 l pots containing forest soil (three plants per pot) and cultivated in four Open–Top Chambers (OTCs; 1.53 m^3^ each) inside a glasshouse (Oliveira et al., 2010). Half of the plants were kept in two OTCs with ambient atmospheric [CO_2_] (380 ± 20 ppm) and the other half was kept in two OTCs under elevated [CO_2_] (760 ± 50 ppm) provided by a cylinder containing 99.8% CO_2_. [CO_2_] inside the chambers was monitored at intervals of 15 min using an infrared gas analyzer (IRGA; WMA 4, PP-Systems Amesbury, MA, USA), coupled to a data logger CR1000 configured using the software LoggerNet 4.0 (Campbell Sci., Logan, UT, USA).

Prior to irrigation treatments, plants remained in the OTCs for 45 days, under the CO_2_ concentration defined for each group, for adaptation. During this period, plants received weekly 100 ml of Vernonia nutrient solution (Cuzzuol et al., 2005) and otherwise, soil was kept moist by water irrigation. For each CO_2_ concentration, plants were then separated into four groups to receive different water replacement treatments, as follows: (i) 100% (control – 100% plants), (ii) 75% (low drought – 75% plants), (iii) 50% (medium drought – 50% plants), and (iv) 25% (severe drought – 25% plants) of the total evapotranspirated water in the previous 48 h, as determined by weight of each plant-pot set. Plants were collected at 0, 9, 18, and 27 days after the beginning of water replacement treatment. Following the 27-day sampling, all plant-soil sets were re-watered to field capacity and a last sampling was done after 5 days. Soil and plant material were submitted to measurements of soil moisture and soil (Ψ_wsoil_), leaf (Ψ_wleaf_), and rhizophore (Ψ_wrhiz_) water potential, leaf gas exchange and determinations of fresh and dry mass of shoots and rhizophores. Rhizophore samples were weighed and frozen in liquid nitrogen for enzyme and carbohydrate analyses.

The experiment was conducted from May to June 2011, under natural photoperiod (11 h–12 h 30 min). Air temperature inside the OTC chambers ranged from 12.2 to 30.2°C and the average temperature was 18°C; relative air humidity varied from 42 to 97%, and PAR (photosynthetic active radiation) ranged approximately from 0.8 to 740 μmol s^-1^ m^-2^ along the day. The experiment was carried out in a completely randomized design and all measurements and extractions were done in triplicate, each one corresponding to two plants.

### Soil Moisture, Dry Biomass, Water Content, and Water Potential

Soil moisture (WCsoil) was measured by gravimetry (Blake, 1965). Water content (%) of aerial organs and rhizophores were determined using the equation WC = (FM–DM)/FM × 100, where WC is water content, FM is the fresh mass, and DM is the dry mass. Dry mass of aerial organs and rhizophores was obtained after drying the tissues at 60°C until constant weight. Leaf water potential (Ψ_wleaf_) was measured using a Scholander-type pressure bomb (model 1000, PMS Instrument, Albany, OR, USA) between 05:00 and 06:00 a.m. The rhizophore osmotic potential was determined in the cell sap using an osmometer, as described in Oliveira et al. (2013). Osmolarity measured in mmol kg^-1^ was transformed into MPa (Y_wrhiz_) using the Van’t Hoff equation, where MPa = mmol kg^-1^ × 2.58 × 10^-3^ (Santa-Cruz et al., 2002).

### Leaf Gas Exchange

Instant measurements of net leaf CO_2_ assimilation rates (*A*), stomatal conductance to water (*g*_s_), and transpiration (*E*) were done at the middle portion of the first leaf with a visible dewlap, under saturating light from 09:00 to 11:00 h, with a 450 mmol m^-2^ s^-1^ PPFD and at 30°C, using a portable photosynthesis system (LI-6400; Li-Cor, Lincoln, NE, USA) as described in Oliveira et al. (2010). The [CO_2_] inside the chamber was the same as in the respective OTC. Instant WUE is defined as the ratio of assimilation rate/transpiration (A/*E*).

### Enzyme Extraction and Assays

Frozen samples of rhizophores (100 mg) were ground in liquid nitrogen and homogenized in 0.05 M McIlvaine buffer (pH 5.5, 1:1, w/v), containing 2 mol m^-3^ EDTA, 2 mol m^-3^ β-mercaptoethanol, 5 mol m^-3^ ascorbic acid, and 10% PVPP (w/w), as described by Asega and Carvalho (2004). Proteins precipitated with (NH_4_)_2_SO_4_ to a final saturation of 80% were re-suspended in a ratio of *ca*. 10 g fresh mass equivalent cm^-3^ in extraction buffer. Enzymes were assayed by incubation of the protein extract with different substrates mixed in the proportion 1:1 (v/ v). All substrates were prepared in 0.05 M McIlvaine buffer with pH 4.5 for 1-FEH and pH 5.5 for 1-SST and 1-FFT activities. The extracts were incubated at 30°C at a final concentration of 0.2 M sucrose (Sigma–Aldrich Co., USA) for 1-SST activity, 0.2 M 1-ketose for 1-FFT activity, and 5% inulin from *C. obovata* for 1-FEH activity. Incubation time was 6 h for 1-SST and 1-FFT and 1 h for 1-FEH. The reactions were stopped by heating the mixture for 5 min at 100°C. Extraction and assay conditions were optimized to assure reliable activity measurements. For the determination of 1-SST, 1-FFT, and 1-FEH activities, samples of the reaction mixtures were diluted 10× and analyzed by HPAEC/PAD using a 2 × 250 mm CarboPac PA-1 column on a Dionex System ICS 3000 (USA). The gradient was established by mixing eluant A (150 mM NaOH) with eluant B (500 mM sodium acetate in 150 mM NaOH) as described in Oliveira et al. (2013). The activities of 1-FEH, 1-SST, and 1-FFT were calculated by directed measurement of fructose, 1-ketose, and nystose, respectively, produced in the reaction mixture, using the external standard method.

### Soluble Carbohydrate Analyses

Carbohydrates were extracted from freeze-dried samples of rhizophores (Portes and Carvalho, 2006). The crude extract was submitted to ethanol precipitation and fructo-oligosaccharide (hexoses, sucrose, and fructans with DP 3–27) and fructo-polysaccharide (mainly fructans with DP 10–60) fractions were separated by centrifugation. Free and combined fructose were measured in the crude extracts and in the separated fractions by a ketose-specific modification of the anthrone reaction (Jermyn, 1956), and used to calculate fructo-oligo:fructo-polysaccharide, while reducing sugars were measured in the fructo-oligosaccharide fraction (Somogyi, 1945). In both reactions, fructose (Sigma–Aldrich Co., USA) was used as standard. Soluble carbohydrates in the oligosaccharide fraction were de-ionized through ion exchange columns, according to Carvalho and Dietrich (1993). Samples containing 400 μg fructose ml^-1^equivalent were analyzed by HPAEC/PAD, using the conditions previously described for 1-FEH analysis.

### Statistical Analyses

The correlation coefficients between Ψ_wsoil_, WCsoil, Ψ_wleaf_, Ψ_wrhiz_, A, *g*_s_, *E*, WUE, and biochemical parameters were calculated using Pearson’s correlation. The statistical significance of the correlation analyses was tested by the independent Student’s *t*-test. In addition, where indicated, one-way ANOVA test was used to verify the statistical significance among the different water treatments and between the two CO_2_ concentrations, using Bioestat 5.0 (Ayres et al., 2007). When significant differences were found, Tukey test was performed at *P* < 0.05. Standard errors of the means (*n* = 3) are also shown. Principal component analysis (PCA) was carried out using the following parameters: net assimilation, transpiration, stomatal conductance, WUE, water potential and biomass of aerial organs and rhizophores, soil moisture, poly and oligosaccharides, reducing sugars, SST and FEH activities. The analysis was done using 100 and 25% plants under both CO_2_ conditions before re-watering. The randomization test (999 permutations) was used to choose the PCA interpretation dimension (*P* < 0.05), using the program PC-ORD 6.0.

## Results

### Plant Water Status, Leaf Gas Exchange, and Plant Biomass

**FIGURE 1:**
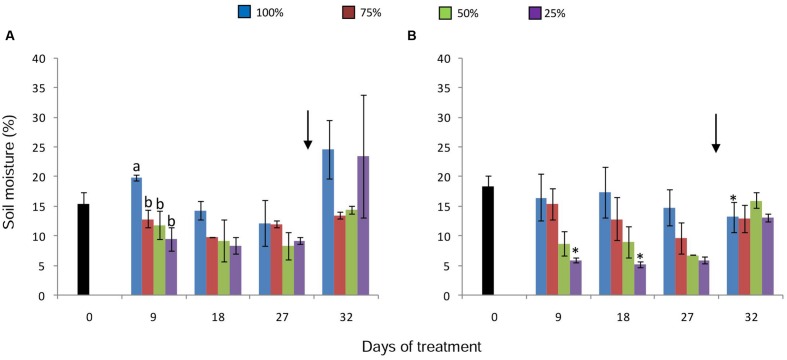
**Soil moisture in pots of *Chrysolaena obovata* at time zero (■) and under different water replacement treatments: 100, 75, 50, and 25%, under ~ 380 ppm **(A)** and ~ 760 ppm **(B)** of CO_2_.** Values are mean ± SE (*n* = 3). Different letters in the graph represent significant differences between water treatments in the same sampling day at *P* > 0.05. * Represents significant differences between CO_2_ concentrations. Arrow indicates the start of re-watering.

The different water replacement treatments imposed on plants resulted in a decrease of soil moisture (**Figures 1A,B**), showing statistical differences between control and treated plants on day 9, under 380 ppm CO_2_ (**Figure 1A**). The same tendency was found on the other sampling dates in both [CO_2_]; however, more distinctly in the 50 and 25% water replacement treatment under high [CO_2_] (**Figure 1B**). The lowest value of soil moisture was found in plant soils under 25% (WCsoil = 5%) and high [CO_2_] on the day 9. Under both [CO_2_], re-watering on the day 27, led to the recovery of soil moisture to values similar to the control.

**FIGURE 2:**
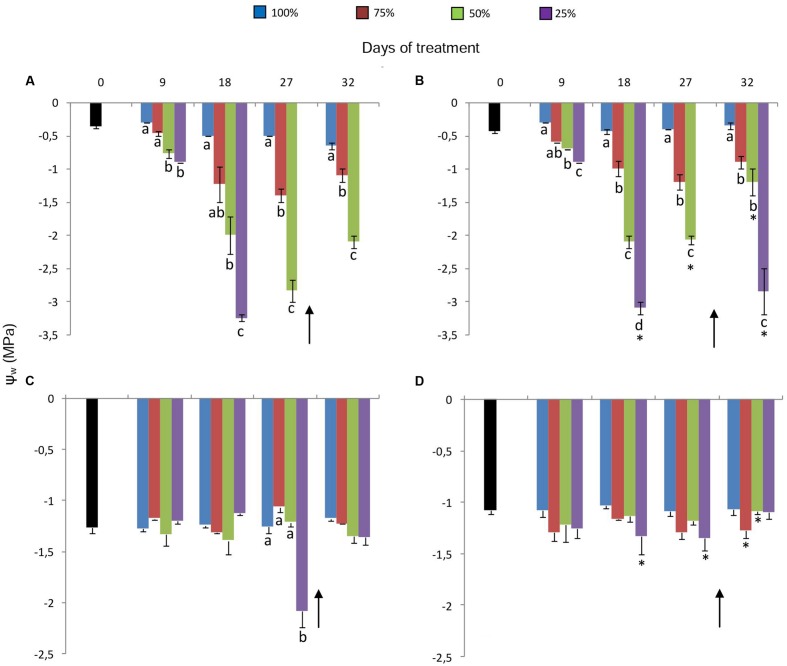
**Leaf (Ψ_wleaf_**A, B**) and rhizophore (Ψ_wrhiz_**C, D**) water potentials in plants of *Chrysolaena obovata* at time zero (■) and under different water replacement treatments: 100, 75, 50, and 25%, under ~ 380 ppm **(A, C)** and ~ 760 ppm **(B, D)** of CO_2_.** Values are mean ± SE (*n* = 3). Different letters represent significant differences between water treatments in the same sampling day at *P* > 0.05. * Represents significant differences between CO_2_ concentrations. Arrow indicates the start of re-watering.

Water replacement treatments caused a decrease in leaf water potential (Ψ_wleaf_) of plants maintained in both 380 ppm (**Figure 2A**) and 760 ppm CO_2_ (**Figure 2B**), corresponding to the water deficit imposed. The decrease, more pronounced in plants under 25%, was first detected on the day 9. After 18 days at 25%, when Ψ_wleaf_ reached very low values (-3.0 MPa) in both [CO_2_], leaves began to senesce, which prevented water potential measurements. As for rhizophores, initial water potential (Ψ_wrhiz_) was ~-1.3 MPa for 380 ppm plants and ~-1.1 MPa for 760 ppm plants (**Figures 2C,D**). A marked decrease in Ψ_wrhiz_ (-2.1 MPa) was only observed in plants under 380 ppm CO_2_ and 25% of water replacement on the day 27 (**Figure 2C**). Re-watering enabled partial recovery of Ψ_wleaf_ and total recovery of Ψ_wrhiz_ in plants under 25% in both [CO_2_].

**FIGURE 3:**
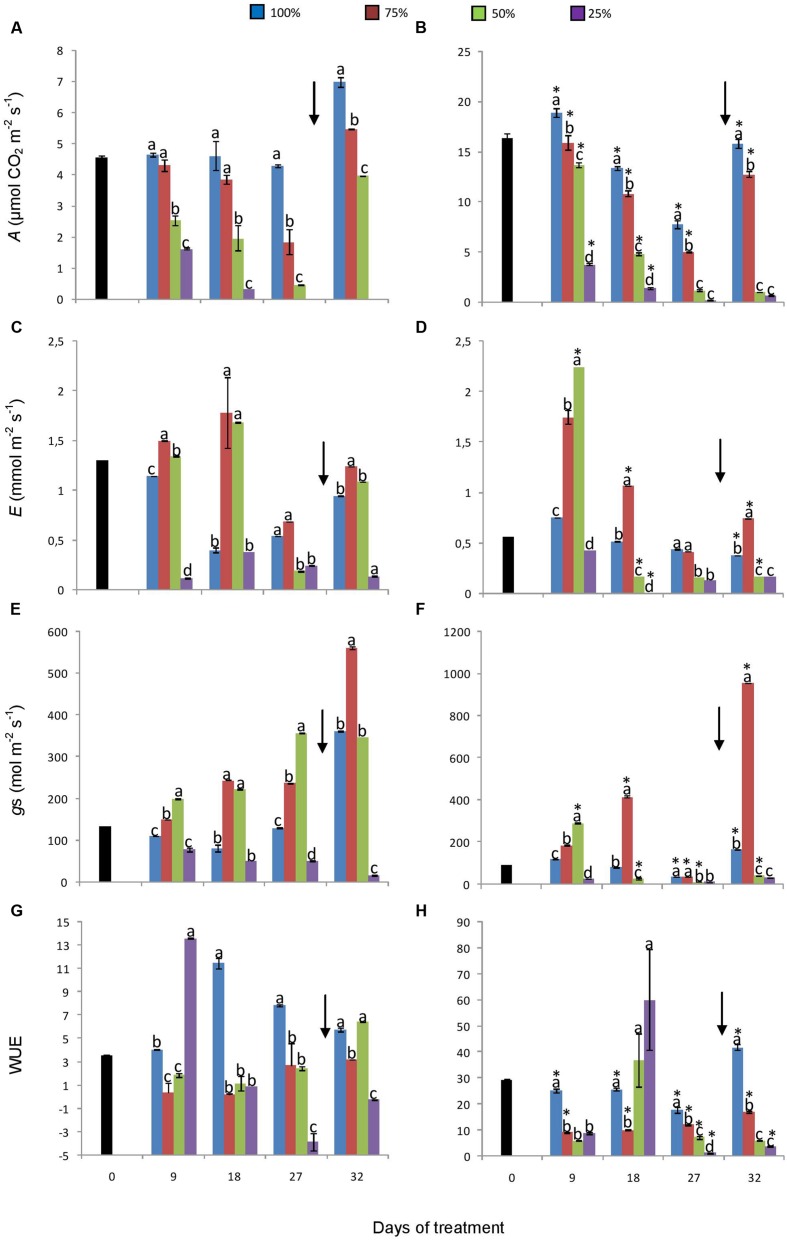
**Net assimilation **(A, B)**, transpiration **(C, D)**, stomatal conductance **(E, F)**, and water use efficiency **(G, H)** in plants of *Chrysolaena obovata* at time zero (■) and under different water replacement treatments: 100, 75, 50, and 25%, under ~ 380 ppm **(A, C, E, G)** and ~ 760 ppm **(B, D, F, H)** of CO_2_.** Values are mean ± SE (*n* = 3). Different letters represent significant differences between water treatments in the same sampling day at *P* > 0.05. * Represents significant differences between CO_2_ concentrations. Arrow indicates the start of re-watering.

Gas exchange parameters such as net CO_2_ assimilation (*A*), stomatal conductance, and WUE were generally higher in plants under 760 ppm CO_2_ (**Figure 3**). In plants under 760 ppm CO_2_ (**Figure 3B**), *A* was four times higher than under 380 ppm at the beginning of the experiment, maintaining higher values of photosynthesis in control and in 75% plants. In both atmospheric [CO_2_], *A* followed the down gradient of water replacement treatments and plants showed negative values on day 27 under 25% and 380 ppm CO_2_ (**Figures 3A,B**). Transpiration (*E*) and stomatal conductance in turn were higher in 75 and 50% plants than in control plants, even after re-watering (**Figures 3C–F**). These three parameters were lower in 25% than in 50 and 75% plants in both [CO_2_]. In plants under 380 ppm CO_2_, the WUE was higher in control plants, except on the day 9, when 25% plants showed higher WUE (**Figure 3G**). Under 760 ppm CO_2_, WUE was also higher in control plants, except on day 18, when 50 and 25% plants presented higher values of WUE (**Figure 3H**). After re-watering, plants under 50 and 75% water replacement at both [CO_2_] showed increases in all parameters, while plants under 25% showed only a slight increase in photosynthetic assimilation and WUE.

**FIGURE 4:**
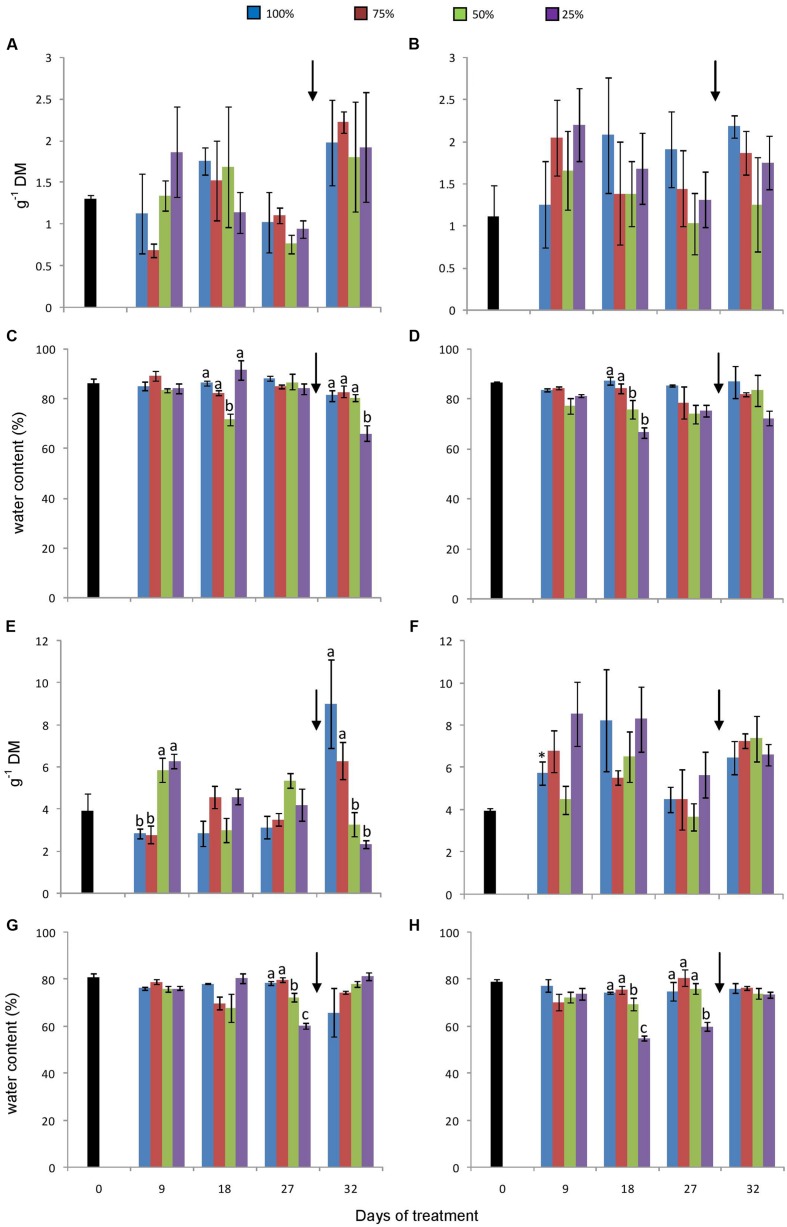
**Dry biomass **(A, B)** and water contents **(C, D)** of aerial organs and dry biomass **(E, F)** and water contents **(G, H)** of rhizophores in plants of *Chrysolaena obovata* at time zero (■) and under different water replacement treatments: 100, 75, 50, and 25%, under ~ 380 ppm **(A, C, E, G)** and ~ 760 ppm **(B, D, F, H)** of CO_2_.** Values are mean ± SE (*n* = 3). Different letters represent significant differences between water treatments in the same sampling day at *P* > 0.05. * Represents significant differences between CO_2_ concentrations. Arrow indicates the start of re-watering.

Water restriction was sufficient to cause changes, although not statistically significant, in aerial biomass, compared to control plants in both [CO_2_] conditions (**Figure 4**). After re-watering, increases in biomass were observed in all water-restriction treatments, although statistically significant in only 75% plants (*P* = 0.0023). Under 760 ppm CO_2_, increases in biomass were observed in the three water-restriction treatments on the day 9 (**Figure 4B**), followed by a decrease until the day 27. Control plants; however, showed a slight tendency to increase dry biomass under this atmospheric condition. Re-watering induced a slight increase in biomass of plants that were subjected to water stress. Even under water restriction, there were no substantial changes in aerial water contents in plants under different treatments, except for 25% plants (severe drought) under 380 ppm CO_2_, on the day 32 (**Figures 4C,D**).

Under ambient CO_2_, slight changes were observed in the rhizophore biomass before re-watering. Following re-watering, rhizophore biomass was higher in 100 and 75% than in 50 and 25% plants, respectively (**Figure 4E**). Under 760 ppm CO_2_, all plants presented a net biomass accumulation when compared to Time 0, which ranged from 63 to 86% on day 32. On day 9, biomass in control plants was statistically higher than it was under 380 ppm CO_2_ (**Figures 4E,F**). Water suppression promoted changes in rhizophore water content in both [CO_2_] (**Figures 4G,H**), and were statistically significant in 25% plants, especially on the day 27. After re-watering, recovery of the moisture content by rhizophores was observed in plants under water stress in both [CO_2_].

### Fructan Metabolism

**FIGURE 5:**
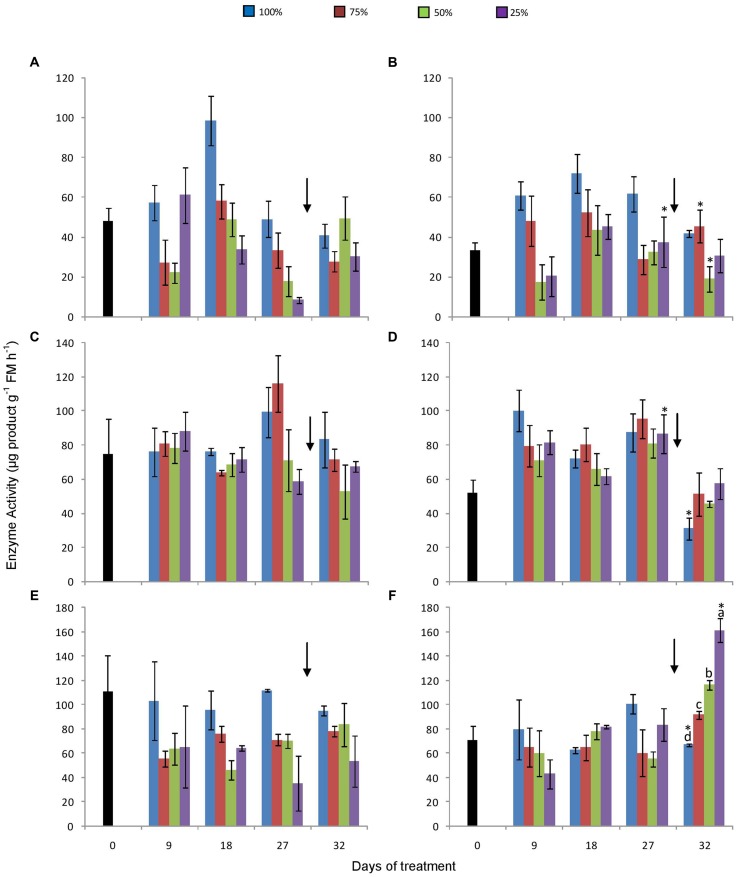
**Activities of 1-SST **(A,B)**, 1-FFT **(C,D)**, and 1-FEH **(E,F)** in rhizophores of plants of *Chrysolaena obovata* at time zero (■) and under different water replacement treatments: 100, 75, 50, and 25%, under ~ 380 ppm **(A, C, E)** and ~ 760 ppm **(B, D, F)** of CO_2_.** Values are mean ± SE (*n* = 3). Different letters represent significant differences between water treatments in the same sampling day at *P* > 0.05. * Represents significant differences between CO_2_ concentrations. Arrow indicates the start of re-watering.

1-SST activity increased in both atmospheric conditions in control plants until the day 18, decreasing afterwards until the end of the experiment (**Figures 5A,B**). However, statistical differences between sampling dates were not found. In general, in all treated plants under 380 ppm CO_2_, 1-SST activity was higher in control plants, and tended to decrease with the decline in water availability. After re-watering, water stressed plants had higher 1-SST activity. These differences, however, were not always statistically significant. Under 760 ppm CO_2_, this tendency was not so obvious, but values were mostly higher in control plants. Re-watering did not affect 1-SST activity in plants under high [CO_2_].

1-FFT activity was not clearly affected by water restriction in any of the CO_2_ treatments (**Figures 5C,D**). After re-irrigation, the activity tended to decrease under water restriction under 380 ppm, while the opposite was detected under 760 ppm CO_2_.

Under ambient [CO_2_], 1-FEH activity was lower, but not significantly different than control plants (**Figure 5E**). Under 760 ppm CO_2_, this tendency was not observed. However, after re-watering the activity increased significantly with the increase in water restriction (**Figure 5F**).

**FIGURE 6:**
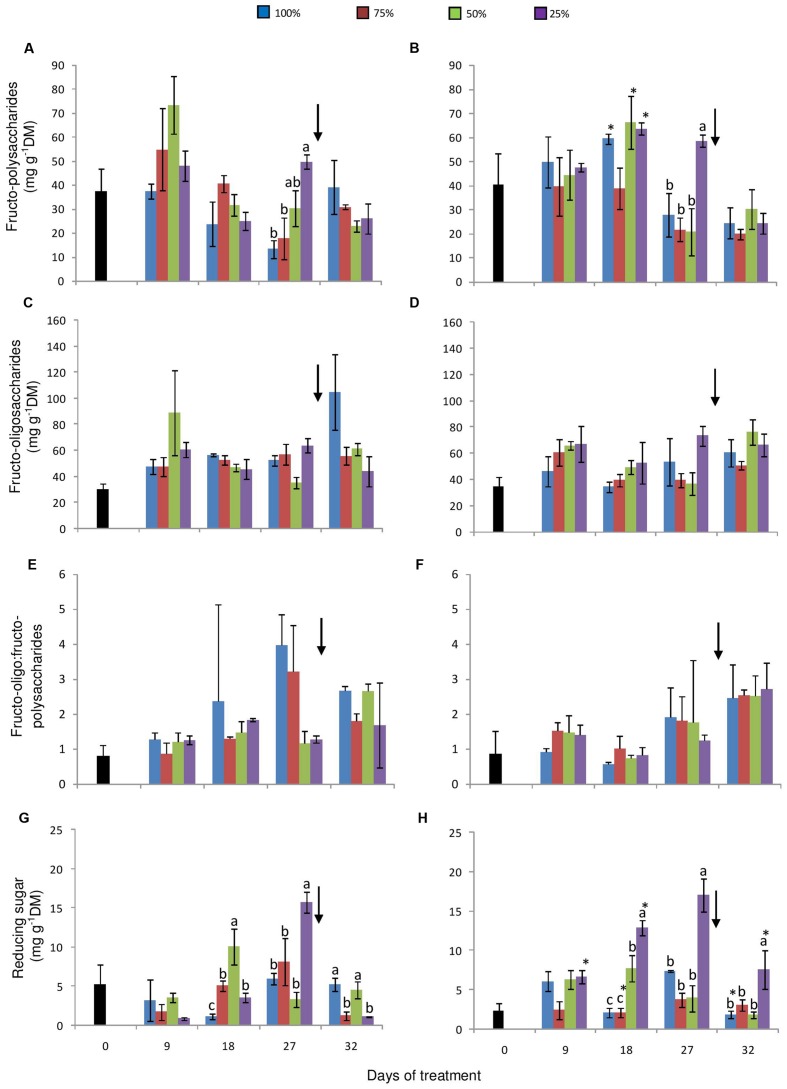
**Fructo-polysaccharide (high DP fructans) **(A, B)** and fructo-oligosaccharide (hexoses, sucrose and low DP fructans) **(C, D)** contents, fructo-oligo:fructo-polysaccharide ratio **(E, F)** and reducing sugar contents **(G, H)** in rhizophores of plants of *Chrysolaena obovata* at time zero (■) and under different water replacement treatments: 100, 75, 50, and 25%, under ~ 380 ppm **(A, C, E, G)** and ~ 760 ppm **(B, D, F, H)** of CO_2_.** Values are mean ± SE (*n* = 3). Different letters represent significant differences between water treatments in the same sampling day at *P* > 0.05. * Represents significant differences between the two CO_2_ concentrations. Arrow indicates the start of re-watering.

Under high [CO_2_] an increase in fructo-polysaccharides was observed only on day 18, in control, 50 and 25% plants. High contents of fructo-polysaccharides were detected in 25% plants under both [CO_2_] on day 27. Also, under 380 ppm, fructans increased gradually with the decrease in water availability (**Figures 6A,B**). Contents of total fructose in the fructo-oligosaccharide fraction were generally constant among the water replacement treatments and between the two [CO_2_] (**Figures 6C,D**). None of the treatments imposed caused considerable changes in the ratio of fructo-oligo:fructo-polysaccharides, except for a higher ratio observed in control and in 75% plants on the day 27, under ambient [CO_2_] (**Figures 6E,F**). However, after re-watering, there was a slight increase in this ratio, especially in plants under a more severe water restriction at 760 ppm CO_2_.

Reducing sugar concentrations in the fructo-oligosaccharide fraction were higher in water-restricted plants under 380 ppm CO_2_ than in control plants, starting from the day 9, with significant differences on the days 18 and 27 (**Figure 6G**). Under high [CO_2_], a similar behavior was observed with marked differences between control and 25% plants on the days 18 and 27 (**Figure 6H**). In both [CO_2_], re-watering led to a decrease in reducing sugars in all water-restriction treatments.

**FIGURE 7:**
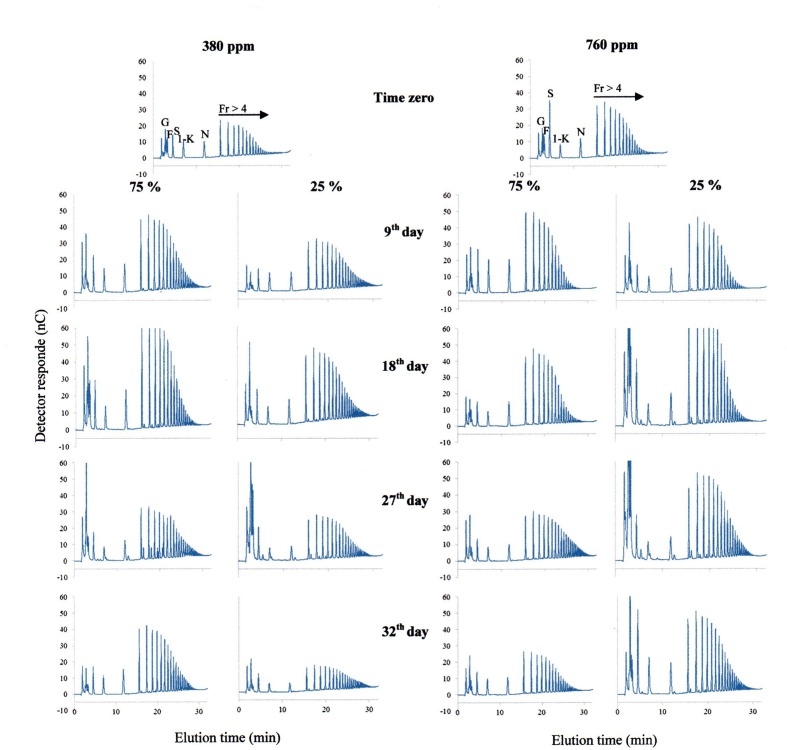
**HPAEC/PAD profiles of fructo-oligossacharides in rhizophores of plants of *Chrysolaena obovata* under 75 and 25% water replacement treatments, under ~ 380 ppm and ~ 760 ppm CO_2_, throughout the experimental period.** G; glucose, F; fructose, S: sucrose, 1-K: 1-ketose, N: nystose, Fr > 4: fructans with DP higher than 4.

Under 380 ppm CO_2_, 75% plants showed an increase in the proportion of FOS (fructo-oligosacharides) in relation to hexoses (glucose and fructose; **Figure 7**). Conversely, 25% plants in the same atmospheric condition showed a twofold increase in hexoses between the days 18 and 27. After re-watering, FOS profile in 25% plants under 380 ppm CO_2_ was similar to the profile observed at time zero in the same atmospheric [CO_2_].

In 75% plants under high [CO_2_], there were no changes in the ratio hexoses: FOS until the day 18. However, on day 18, there was an increase in the proportion of fructans with medium-chain sizes. In 25% plants, there was a considerable increase in the proportion of hexoses from the day 18. This increase in hexoses still remained after re-watering, without changes in FOS (**Figure 7**).

**FIGURE 8:**
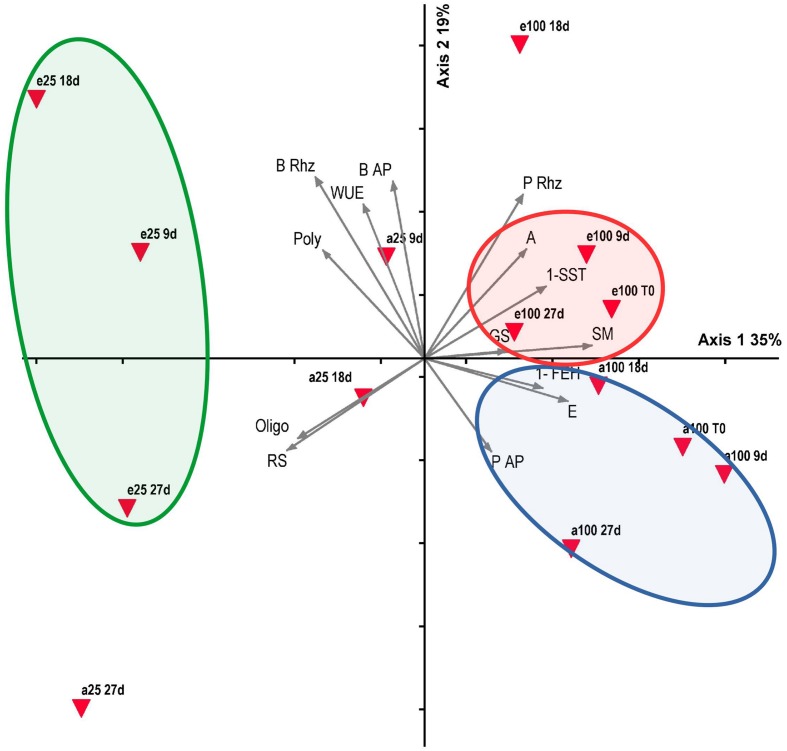
**Principal component analysis (PCA) bi-plot of physiological and biochemical parameters of *Chrysolaena obovata* under ~ 380 ppm **(a)** and ~ 760 ppm (e) CO_2_, and 100% and 25% water replacement treatments.** Triangles represent CO_2_, water replacement treatments (100, 25) and sampling days (T0, 9, 18, and 27 days), and vectors represent analyzed parameters: net assimilation (A), transpiration (E), stomatal conductance (GS), water use efficiency (WUE), water potential of aerial organs (P AP) and rhizophores (P Rhz), soil moisture (SM), fructo-polysaccharides (Poly), fructo-oligosaccharides (Oligo), 1-SST, 1-FEH, reducing sugars (RS), biomass of aerial organs (B AP) and rhizophores (B Rhz).

Correlation coefficients and their significance are presented in Supplementary Table S1 and the most noteworthy are shown in the Section “Discussion”. PCA summarized 54.8% of the total data variability on the two first axes (**Figure 8**), which were considered significant at the randomization test (*P* < 0.005). All samplings at 100% water replacement in both [CO_2_] concentrations positioned on the positive side of axis 1, which indicated a correlation with net assimilation (*A*), rhizophore water potential (P Rhiz) (*r* > 0.5), SST, and FEH activities (*r* > 0.6), transpiration (E) (*r* > 0.7) and soil moisture (SM; *r* = 0.9). Conversely, on the negative side of axis 1, all samplings of 25% water replacement in both [CO_2_] concentrations correlated with fructo-polysaccharides (Poly) and rhizophore biomass (B Riz) (*r* > -0.5), fructo-oligosaccharides (Oligo) (*r* > -0.6) and reducing sugars (RS) (*r* > -0.7). Concerning axis 2, all samplings under high [CO_2_], except A25 9d (25% plants under ambient CO_2_, day 9) correlated with WUE and rhizophore water potential (*r* > 0.6), and aerial (BAP) and rhizophore biomass (*r* > 0.7). All samplings under ambient CO_2_, except E25 27 days (25% plants under 760 ppm, day 27), were located on the negative side of axis 2, however not showing high correlation with the parameters. PCA axes indicated a relationship of CO_2_ atmospheric conditions and water availability on physiological and biochemical responses of *C. obovata.*

## Discussion

In the present study, significant decreases in volumetric content and in the free energy state of water in soil and plants, caused by different levels of water replacement, affected physiological and biochemical processes such as photosynthesis and fructan metabolism in plants of *C. obovata* under 380 and 760 ppm CO_2_. Changes in biomass were not clearly detected due to the short experimental period. However, plants under severe drought in both [CO_2_] recovered the water content after re-watering.

As most Cerrado species, *C. obovata* is a slow-growing species, although high relative growth rates were detected after 30 days of high [CO_2_] exposition, and biomass increased only after longer periods under this condition. Additionally, increased photosynthesis rates were detected under elevated [CO_2_] and regular watering (Oliveira et al., 2010). In the present study, photosynthesis was dependent on water availability and decreased significantly in plants under water suppression in relation to control plants in both atmospheric [CO_2_]. In fact, in plants under water deficit, gas exchange was limited or totally inhibited due to the senescence of aerial organs. In addition, high [CO_2_] minimized the short-time effects of water stress on photosynthesis, since rates were kept high under low (75%) water deficit and could be detected even in plants under severe drought (25%). In 380 ppm CO_2_, photosynthesis was detected under severe drought only until day 18. Among the earliest changes in plants exposed to drought is the reduction of water loss during transpiration, by regulation of stomatal opening, this being a major indirect factor reducing photosynthesis that significantly limits carbon assimilation and growth (Burgess, 2006). In the present study, the most pronounced decrease in stomatal conductance was observed in 25% plants under 380 and 760 ppm [CO_2_], data that correlated significantly with soil moisture (WCsoil ×*g*_s_, *r* = 0.96; *P* = 0.001 and *r* = 0.98, *P* = 0.0006, respectively). Therefore, the decrease in soil moisture was the main factor limiting photosynthesis in plants under these conditions, regardless of the [CO_2_].

A number of studies suggested that plants under high [CO_2_] lose water more slowly under water stress when compared to irrigated plants, due to lower stomatal conductance and transpiration rates (Robredo et al., 2007, and the references therein). In *C. obovata* grown under high [CO_2_], stomatal conductance and transpiration were correlated significantly with the Ψ_wrhiz_ in 25% plants. Water potential in rhizophores was similar in all water regimes throughout the experimental period (Ψ_wrhiz_ ×*g*_s_, *r* = 0.98; *P* = 0.0001, Ψ_wrhiz_ × *E*, *r* = 0.88; *P* = 0.04), whereas under 380 ppm, 25% plants exhibited no correlation between gas exchange and Ψ_wrhiz_, and declining in Ψ_wrhiz_ was detected after the day 18. These results differ from those reported by Oliveira et al. (2013) for *V. discolor*. In the later species, *E* and *gs* were higher in plants under high [CO_2_] and drought, demonstrating that, in spite of several similarities such as fructan metabolism and that they coexist in Cerrado, *C. obovata* and *V. discolor* demonstrate different gas exchange features under the same stress conditions. While in *V. discolor*, gas exchange seems to be more determined by water availability, in *C. obovata*, gas exchange was modulated by high [CO_2_], since plants under water restriction were able to maintain water status.

Previous studies showed that during long periods under water stress, rhizophores of *C. obovata* demonstrated a high water holding capacity, possibly related to the ability of fructans to act in osmoregulation. In fact, in plants under 380 ppm, water retention was accompanied by changes in fructan metabolism leading to increases in hexoses, sucrose and small-chain fructans, as previously reported by Dias-Tagliacozzo et al. (2004) and Garcia et al. (2011) in plants of *C. obovata* subjected to drought.

Regarding fructosyltransferases, changes were more considerable in 1-SST than in 1-FFT activity, highlighted by a decrease in 1-SST in plants under water restriction in both [CO_2_], despite non-significant differences from the control. Portes and Carvalho (2006) reported a decrease in 1-SST activity in *C. obovata* during natural sprouting, dormancy and induced sprouting, stages in which the photosynthetic apparatus was absent or insufficient to provide assimilates for fructan synthesis. In fact, in the present study we found a significant positive correlation between decrease in photosynthesis and 1-SST activity at different levels of water replacement in both [CO_2_] (see Correlation Supplementary Table S1 in Supplementary Material), similar to results obtained with *V. discolor* (Oliveira et al., 2013) and other fructan-accumulating species (De Roover et al., 1999; Asega and Carvalho, 2004; Joudi et al., 2012; Rigui et al., 2015).

After re-watering, plants under 760 ppm CO_2_ demonstrated increases in 1-FEH activity that were inversely proportional to the intensity of water restriction, whereas in plants under 380 ppm CO_2_ this was not observed. The increased proportion of hexoses in relation to FOS, clearly observed on the days 18 and 27 of water restriction, was more pronounced under high [CO_2_], indicating the interactive effect of both factors (CO_2_ concentration and water restriction) on fructan metabolism. Five days after re-watering (day 32), 25% plants under high [CO_2_] grew new sprouts, possibly relying on the products of 1-FEH activity as carbon source. The association of sprouting and fructan mobilization by 1-FEH has been described for *C. obovata*, as well as other Asteraceae and Poaceae (Carvalho et al., 2007, and the references therein; Joudi et al., 2012).

Invertases generating a pool of hexoses under high [CO_2_] is an important aspect of an early response to lower water availability. Invertase activity may explain the decrease of the sucrose peak in 25% plants on day 9. The higher sucrose peak observed at time zero in comparison to plants under 380 ppm CO_2_ could be a result of the higher carbon intake under high [CO_2_]. Interactive effects of high [CO_2_] and extreme climate conditions were reported for fructan-accumulating grasses and shown to contribute to increases in hexoses, sucrose, and fructans (AbdElgawad et al., 2014).

The most significant correlations between WC soil, Ψ_wleaf_ and Ψ_wrhiz_ and the physiological and biochemical parameters in ambient CO_2_ (Ψ_wleaf_ ×*A*, *r* = 0.84; *P* = 0.03, reducing sugar × Ψ_wrhiz_, *r* = -0.91, *P* = 0.01, Ψ_wleaf_ × FFT, *r* = 0.85; *P* = 0.03, for example) and in high [CO_2_] (FFT × WC soil, *r* = 0.89; *P* = 0.01, Ψ_wleaf_ ×*g_s_*, *r* = 0.74, *P* = 0.08, Ψ_wleaf_ ×*E*, *r* = 0.97, *P* = 0.0006, for example), were verified in 25% plants.

In contrast to the results in 25% plants, a gradual decrease in water availability and maintenance of water status was observed in the 50 and 75% moisture treatments. Furthermore, fructan contents and the activity of fructan-metabolizing enzymes in rhizophores were also maintained close to values detected in control plants. This suggests a process of acclimatization of plants to these two water conditions, however more efficiently under high [CO_2_]. According to Larcher (2003), if the intensity of a stressing factor does not change, repairing processes such as the synthesis of protective substances are quickly initiated. This situation leads to a resistance phase in which, under continuous stress, resistance increases. Due to the improved stability under continuous stress, plants adapt to the new conditions, a situation that seems to have occurred in plants of *C. obovata* under the intermediate water treatments.

PCA confirms what was pointed out in the results indicating the direct influence of the elevated [CO_2_] on parameters related to carbon gain (A) and maintenance (1-SST), contrary to ambient [CO_2_], which influenced mostly parameters related to water loss (E) and fructan breakdown (1-FEH). In plants under water restriction, high [CO_2_] affected processes that favor the maintenance of water status such as WUE and the production of fructo-oligosaccharides and reducing sugars, the two later acting on osmoregulation to prevent water loss.

Under the predicted climate change scenarios of increase in [CO_2_] and changes in rain distribution, mainly leading to long drought periods in South America, species capable of modulating carbon gain and water loss, such as *C. obovata*, seem to be more fitted to the new environment. This study demonstrates that high [CO_2_] reduces the negative effects of drought in *C. obovata*, mainly by maintaining a high carbon intake through photosynthesis and adjusting fructan metabolism in response to these environmental factors. These features comprise important strategies for species survival in the predicted climate scenario. Considering that the herbaceous layer is the most biodiverse rich in the Cerrado, this study also highlights the importance of adding new information of a native fructan-accumulating species from a relevant functional group, concerning responses to climate changes.

## Author Contributions

VO, ES, and MC designed the research. VO conducted the experiments and performed the statistical analysis. VO, ES, and MC analyzed the data and wrote the manuscript. All authors have read, revised and approved the final manuscript.

## Conflict of Interest Statement

The authors declare that the research was conducted in the absence of any commercial or financial relationships that could be construed as a potential conflict of interest. The reviewer AM-B and handling Editor declared their shared affiliation, and the handling Editor states that the process nevertheless met the standards of a fair and objective review.

## References

[B1] AbdElgawad H. Peshev D. Zinta G. Van den Ende W. Janssens I. A. Asard H. (2014). Climate extreme effects on the chemical composition of temperate grassland species under ambient and elevated CO2: a comparison of fructan and non-fructan accumulators. *PLoS ONE* 9:e92044 10.1371/journal.pone.0092044 PMC3966776 24670435

[B2] Allen L. H. Jr. (1994). “Carbon dioxide increase: direct impacts on crops and indirect effects mediated through anticipated climate changes,” in *Physiology and Determination of Crop Yield* eds Boote K. J. Bennett J. M. Sinclair T. R. Paulse G. M. (Madison, WI: Soil Science Society of America), 425–459.

[B3] Ameye M. Wertin T. M. Bauweraerts I. Mcguire M. A. Teskey R. O. Steppe K. (2012). The effect of induced heat waves on *Pinus taeda* and *Quercus rubra* seedlings in ambient and elevated CO2 atmospheres. *New Phytol.* 196 448–461. 10.1111/j.1469-8137.2012.04267.x 22897414

[B4] Asega A. F. Carvalho M. A. M. (2004). Fructan metabolizing enzymes in rhizophores of *Vernonia herbacea* upon excision of aerial organs. *Plant Physiol. Biochem.* 42 313–319. 10.1016/j.plaphy.2004.02.005 15120116

[B5] Asega A. F. Nascimento J. R. O. Carvalho M. A. M. (2011). Increased expression of fructan 1-exohydrolase in rhizophores of *Vernonia herbacea* during sprouting and exposure to low temperature. *J. Plant Physiol.* 168 558–565. 10.1016/j.jplph.2010.09.002 20950891

[B6] Ayres M. Ayres M. Jr. Ayres D. L. Santos A. A. S. (2007). *BioEstat 5.0: Aplicações Estatísticas nas Áreas de Ciências Biológicas e Médicas.* Belém: Sociedade Civil Mamirauá.

[B7] Bassow S. L. McConnaughay K. D. M. Bazzaz F. A. (1994). The response of temperate tree seedlings grown in elevated CO2 to extreme temperature events. *Ecol. Appl.* 4 593–603. 10.2307/1941960

[B8] Bauweraerts I. Wertin T. M. Ameye M. Mcguire M. A. Teskey R. O. Steppe K. (2013). The effect of heat waves, elevated CO2 and low soil water availability on northern red oak (*Quercus rubra* L.) seedlings. *Glob. Change Biol.* 19 517–528. 10.1111/gcb.12044 23504789

[B9] Blake G. R. (1965). “Bulk density,” in *Methods of Soil Analysis* eds Black C. A. Evans D. D. White J. L. Ensminger L. E. Clark F. E. (Madison, WI: American Society of Agronomy, Inc.), 374–390.

[B10] Burgess S. S. O. (2006). Facing the challenge if seasonally dry environments. *Physiol. Plant.* 127 339–342. 10.1111/j.1399-3054.2006.00726.x

[B11] Carvalho M. A. M. Asega F. A. Figueiredo-Ribeiro R. C. L. (2007). “Fructans in Asteraceae from Brazilian cerrado,” in *Recent Advances in Fructooligosaccharides Research* eds Norio S. Noureddine B. Shuichi O. (Kerala: Research Signpost), 69–91.

[B12] Carvalho M. A. M. Dietrich S. M. (1993). Variation in fructan content in the underground organs of *Vernonia herbacea* (Vell.) Rusby at different phenological phases. *New Phytol.* 123 735–740. 10.1111/j.1469-8137.1993.tb03784.x

[B13] Carvalho M. A. M. Pinto M. M. Figueiredo-Ribeiro R. C. L. (1998). Inulin production by *Vernonia herbacea* as influenced by mineral fertilization and time of harvest. *Braz. J. Bot.* 21 275–280. 10.1590/S0100-84041998000300006

[B14] Centritto M. Lee H. S. J. Jarvis P. G. (1999). Increased growth in elevated [CO2]: an early, short-term response? *Glob. Change Biol.* 5 623–633. 10.1046/j.1365-2486.1999.00263.x

[B15] Chaves M. M. Pereira J. S. (1992). Water stress, CO2 and climate change. *J. Exp. Bot.* 43 1131–1139. 10.1093/jxb/43.8.1131

[B16] Coleman J. S. Rochefort L. Bazzaz F. A. Woodward F. I. (1991). Atmospheric CO2, plant nitrogen status and the susceptibility of plants to an acute increase in temperature. *Plant Cell Environ.* 14 667–674. 10.1111/j.1365-3040.1991.tb01539.x

[B17] Curtis P. S. Wang X. (1998). A meta-analysis of elevated CO2 effects on woody plant mass, form and physiology. *Oecologia* 113 299–313. 10.1007/s004420050381 28307814

[B18] Cuzzuol G. R. F. Carvalho M. A. M. Zaidan L. B. P. Furlan P. R. (2005). Soluções nutritivas para o cultivo e a produção de frutanos em plantas de *Vernonia herbacea*. *Pesq. Agropec. Bras.* 40 911–917. 10.1590/S0100-204X2005000900011

[B19] De Roover J. Van den Branden K. Van Laere A. Van den Ende W. (2000). Drought induces fructan synthesis and 1-SST (sucrose:sucrose fructosyltransferase) in roots and leaves of chicory seedlings (*Cichorium intybus* L.). *Planta* 210 808–814. 10.1007/s004250050683 10805453

[B20] De Roover J. Van Laere A. Van den Ende W. (1999). Effect of defoliation on fructan pattern and fructan metabolizing enzymes in young chicory plants (*Cichorium intybus*). *Physiol. Plant.* 106 158–163. 10.1034/j.1399-3054.1999.106202.x

[B21] De Souza A. P. Cocuron J.-P. Garcia A. C. Alonso A. P. Buckeridge M. S. (2015). Changes in whole-plant metabolism during the grain-filling stage in sorghum grown under elevated CO2 and drought. *Plant Physiol.* 169 1755–1765. 10.1104/pp.15.01054 26336093 PMC4634081

[B22] Dias B. F. S. (1992). *Alternativas de Desenvolvimento dos Cerrados: Manejo e Conservação dos Recursos Naturais Renováveis.* Brasília: Funatura.

[B23] Dias-Tagliacozzo G. M. Itaya N. M. Carvalho M. A. M. Figueiredo-Ribeiro R. C. L. Dietrich S. M. C. (2004). Fructans and water suppression on intact and fragmented rhizophores of *Vernonia herbacea*. *Braz. Arch. Biol. Technol.* 47 363–373. 10.1590/S1516-89132004000300005

[B24] Duan H. Duursma R. Huang G. Smith R. A. Choat B. O’Grady A. P. (2014). Elevated [CO2] does not ameliorate the negative effects of elevated temperature on drought-induced mortality in *Eucalyptus radiata* seedlings. *Plant Cell Environ.* 37 1598–1613. 10.1111/pce.12260 24372529

[B25] Earth System Research Laboratory [ESRL] (2015). *Earth System Research Laboratory.* Available at: http://www.esrl.noaa.gov/ (accessed February 2015).

[B26] Edelman J. Jefford T. G. (1968). The mechanism of fructosan metabolism in higher plants as exemplified in *Helianthus tuberosus*. *New Phytol.* 67 517–531. 10.1111/j.1469-8137.1968.tb05480.x

[B27] Field C. B. Chapin S. F. Matson P. A. Mooney A. (1992). Responses of terrestrial ecosystems to the changing atmosphere: a resource-based approach. *Annu. Rev. Ecol. Syst.* 23 201–235. 10.1146/annurev.es.23.110192.001221

[B28] Franco A. C. (1998). Seasonal patterns of gas exchange, water relations and growth of *Roupala montana*, an evergreen savanna species. *Plant Ecol.* 136 69–76. 10.1023/A:1009763328808

[B29] Franco A. C. (2002). “Ecophysiology of woody plants,” in *The Cerrados of Brazil: Ecology and Natural History of a Neotropical Savanna* eds Oliveira P. S. Marquis R. J. (New York, NY: Columbia University Press), 178–197.

[B30] Franco A. C. Rossatto D. R. Silva L. C. R. Ferreira C. S. (2014). Cerrado vegetation and global change: the role of functional types, resource availability and disturbance in regulating plant community responses to rising CO2 levels and climate warming. *Theor. Exp. Plant Physiol.* 26 19–38. 10.1007/s40626-014-0002-6

[B31] Garcia P. M. A. Asega A. F. Silva E. A. Carvalho M. A. M. (2011). Effect of drought and re-watering on fructan metabolism in *Vernonia herbacea* (Vell.) Rusby. *Plant Physiol. Biochem.* 49 664–670. 10.1016/j.plaphy.2011.03.014 21531568

[B32] Garcia P. M. A. Hayashi A. H. Silva E. A. Figueiredo-Ribeiro R. C. L. Carvalho M. A. M. (2015). Structural and metabolic changes in rhizophores of the Cerrado species *Chrysolaena obovata* (Less.) Dematt. as influenced by drought and re-watering. *Front. Plant Sci.* 6:721 10.3389/fpls.2015.00721 PMC4585265 26442035

[B33] Gunderson C. A. Wullschleger S. D. (1994). Photosynthetic acclimation in trees to rising atmospheric CO2: a broader perspective. *Photosynth. Res.* 39 369–388. 10.1007/BF00014592 24311130

[B34] Hare P. D. Cress W. A. Van Staden J. (1998). Dissecting the roles of osmolyte accumulation during stress. *Plant Cell Environ.* 21 535–553. 10.1046/j.1365-3040.1998.00309.x

[B35] Hendry G. A. F. Wallace R. K. (1993). “The origin, distribution, and evolutionary significance of fructans,” in *Science and Technology of Fructans* eds Suzuki M. Chatterton N. J. (Boca Raton, FL: CRC Press), 119–139.

[B36] Incoll L. D. Neales T. F. (1970). The stem as a temporary sink before tuberization in *Helianthus tuberosus*. *J. Exp. Bot.* 21 469–476. 10.1093/jxb/21.2.469

[B37] IPCC (2013). “Summary for policymakers,” in *Climate Change 2013: The Physical Science Basis. Contribution of Working Group I to the Fifth Assessment Report of the Intergovernmental Panel on Climate Change* eds Stocker T. F. Qin D. Plattner G.-K. Tignor M. Allen S. K. Boschung J. (Cambridge: Cambridge University Press), 1–30.

[B38] Isejima E. M. Figueiredo-Ribeiro R. C. L. (1993). Fructan variations in tuberous roots of *Viguiera discolor* Baker (Asteraceae): the influence of phenology. *Plant Cell Physiol.* 34 723–727.

[B39] Jermyn M. A. (1956). A new method for the determination of ketohexoses in the presence of aldohexoses. *Nature* 177 38–39. 10.1038/177038a0

[B40] Joudi M. Ahmadi A. Mohamadi V. Abbasi A. Vergauwen R. Mohammadi H. (2012). Comparison of fructan dynamics in two wheat cultivars with different capacities of accumulation and remobilization under drought stress. *Physiol. Plant.* 144 1–12. 10.1111/j.1399-3054.2011.01517.x 21895669

[B41] Kimball B. A. Morris C. F. Pinter P. J. Jr. Wall G. W. Hunsaker D. J. Adamsen F. J. (2001). Elevated CO2, drought and soil nitrogen effects on wheat grain quality. *New Phytol.* 150 295–303. 10.1046/j.1469-8137.2001.00107.x

[B42] Larcher W. (2003). *Physiological Plant Ecology: Ecophysiology and Stress Physiology of Functional Groups.* New York, NY: Springer.

[B43] Long S. P. (1991). Modification of the response of photosynthetic productivity to rising temperature by atmospheric CO2 concentrations: has its importance been underestimated? *Plant Cell Environ.* 14 729–739. 10.1111/j.1365-3040.1991.tb01439.x

[B44] Luo Y. Mooney H. A. (1999). *Carbon Dioxide and Environmental Stress.* San Diego, CA: Academic Press.

[B45] Mantovani W. Martins F. R. (1988). Variações fenológicas das espécies do cerrado da Reserva Biológica de Moji-Guaçú, Estado de São Paulo. *Braz. J. Bot.* 11 101–112. 10.1590/S0034-71082000000100016

[B46] Naudts K. Van Den Berge J. Farfan E. Rose P. AbdElgawad H. Ceulemans R. (2014). Future climate alleviates stress impact on grassland productivity through altered antioxidant capacity. *Environ. Exp. Bot.* 99 150–158. 10.1016/j.envexpbot.2013.11.003

[B47] Oliveira V. F. Silva E. A. Zaidan L. B. P. Carvalho M. A. M. (2013). Effects of elevated CO2 concentration and water deficit on fructan metabolism in *Viguiera discolor* Baker. *Plant Biol.* 15 471–482. 10.1111/j.1438-8677.2012.00654.x 22882384

[B48] Oliveira V. F. Zaidan L. B. P. Braga M. R. Aidar M. P. M. Carvalho M. A. M. (2010). Elevated CO2 atmosphere promotes plant growth and inulin production in the cerrado species. *Funct. Plant Biol.* 37 223–231. 10.1071/FP09164

[B49] Pontis H. G. (1989). Fructans and cold stress. *J. Plant Physiol.* 134 148–150. 10.1016/S0176-1617(89)80047-1

[B50] Portes M. T. Carvalho M. A. M. (2006). Spatial distribution of fructans and fructan metabolizing enzymes in rhizophores of *Vernonia herbacea* (Vell.) Rusby (Asteraceae) in different developmental phases. *Plant Sci.* 170 624–633. 10.1016/j.plantsci.2005.10.017

[B51] Portes M. T. Figueiredo-Ribeiro R. C. L. Carvalho M. A. M. (2008). Low temperature and defoliation affect fructan metabolizing enzymes in different regions of the rhizophores of *Vernonia herbacea*. *J. Plant Physiol.* 165 1572–1581. 10.1016/j.jplph.2008.01.004 18342987

[B52] Quesada C. A. Hodnett M. G. Breyer L. M. Santos A. J. B. Andrade S. Miranda H. S. (2008). Seasonal variations in soil water in two woodland savannas of central Brazil with different fire history. *Tree Physiol.* 28 405–415. 10.1093/treephys/28.3.405 18171664

[B53] Reddy A. Rasineni G. Raghavendra A. (2010). The impact of global elevated CO2 concentration on photosynthesis and plant productivity. *Curr. Sci.* 99 46–57.

[B54] Rigui A. P. Gaspar M. Oliveira V. F. Purgatto E. Carvalho M. A. M. (2015). Endogenous hormone concentrations correlate with fructan metabolism throughout the phenological cycle in *Chrysolaena obovata*. *Ann. Bot.* 115 1177–1190. 10.1093/aob/mcv053 25921788 PMC4648463

[B55] Robredo A. Pérez-López U. Maza H. S. González-Moro B. Lacuesta M. Mena-Petite A. (2007). Elevated CO2 alleviates the impact of drought on barley improving water status by lowering stomatal conductance and delaying its effects on photosynthesis. *Environ. Exp. Bot.* 59 252–263. 10.1016/j.envexpbot.2006.01.001

[B56] Roden J. S. Ball M. C. (1996). The effect of elevated [CO2] on growth and photosynthesis of two eucalyptus species exposed to high temperatures and water deficits. *Plant Physiol.* 111 909–919. 10.1104/pp.111.3.909 12226337 PMC157910

[B57] Rogers H. H. Sionit N. Cure J. D. Smith H. M. Binham G. E. (1984). Influence of elevated CO2 on water relations of soybeans. *Plant Physiol.* 74 233–238. 16663403 10.1104/pp.74.2.233 PMC1066661

[B58] Santa-Cruz A. Martinez-Rodriguez M. M. Perez-Alfocea F. Romero-Aranda R. Bolarin M. C. (2002). The rootstock effect on the tomato salinity response depends on the shoot genotype. *Plant Sci.* 162 825–831. 10.1016/S0168-9452(02)00030-4

[B59] Saxe H. Ellksworth D. S. Heath J. (1998). Tansley review: tree and forest functioning in an enriched CO2 atmosphere. *New Phytol.* 139 395–436. 10.1046/j.1469-8137.1998.00221.x

[B60] Solomon S. Plattnerb G.-K. Knuttic R. Friedlingsteind P. (2009). Irreversible climate change due to carbon dioxide emissions. *Proc. Natl. Acad. Sci. U.S.A.* 106 1704–1709. 10.1073/pnas.0812721106 19179281 PMC2632717

[B61] Somogyi M. (1945). A new reagent for the determination of sugars. *J. Biol. Chem.* 160 61–63.

[B62] Stitt M. Krapp A. (1999). The interaction between elevated carbon dioxide and nitrogen nutrition: the physiological and molecular background. *Plant Cell Environ.* 22 583–621. 10.1046/j.1365-3040.1999.00386.x

[B63] Tognetti J. A. Salerno G. L. Crespi M. D. Pontis H. G. (1990). Sucrose and fructan metabolism of different wheat cultivars at chilling temperature. *Physiol. Plant.* 78 554–559. 10.1111/j.1399-3054.1990.tb05241.x

[B64] Vandoorne B. Mathieu A.-S. Van den Ende W. Vergauwen R. Périlleux C. Javaux M. (2012). Water stress drastically reduces root growth and inulin yield in *Cichorium intibus* (vas. Sativum) independently of photosynthesis. *J. Exp. Bot.* 12 4359–4373. 10.1093/jxb/ers095 22577185 PMC3421980

[B65] Wiemken A. Frehner M. Keller F. Wagner W. (1986). Fructan metabolism, enzymology and compartmentation. *Curr. Top. Plant Biochem. Physiol.* 5 17–37.

[B66] Wiltshire A. Gornall J. Booth B. Dennis E. Falloon P. Kay G. (2013). The importance of population, climate change and CO2 plant physiological forcing in determining future global water stress. *Glob. Environ. Change* 23 1083–1097. 10.1016/j.gloenvcha.2013.06.005

