# Supplementary material for: Elevated CO2 Atmosphere Minimizes the Effect of Drought on the Cerrado Species Chrysolaena obovata
Source: Front Plant Sci. 2016 Jun 14;7:810. doi: 10.3389/fpls.2016.00810 (PMC4905961; doi:10.3389/fpls.2016.00810)
Supplement: Supplementary file 1 [file Table_1.PDF]

Table S1. Correlation coefficients between WC soil, Ψwaerial, Ψwrz, *A* and WUE and physiological and biochemical parameters in plants of *Chrysolaena obovata* under different levels of water replacement (100%, 75%, 50% e 25%). Correlation coefficients were shown only when R ≥ 0.5; * *P*<0,05; ** *P*<0,01; *** *P*<0,001.

		**WC soil**		**Ψwaerial**		**Ψwrz**		***A***		**WUE**	
		**380**	**760**	**380**	**760**	**380**	**760**	**380**	**760**	**380**	**760**
100% plants	**A**	0.79	0.66	0.60							
	**E**	0.66		0.91**	0.81	-0.97***			0.90**	-0.96**	
	**gs**		0.58		0.55	-0.59		-0.53	0.99***		
	**WUE**	-0.58	0.98***	-0.87*		0.98***			0.80*		
	**Aerial Biomass**					0.57	0.58	0.51	-0.74	0.64	
	**WCaerial**	-0.93 ***	-0.64	-0.82*		0.60		-0.95**	-0.41		
	**RZ Biomass**						0.95**				
	**WCrhizophore**	-0.52	0.57				-0.52		0.72		
	**1-SST**		-0.52				0.58	0.69		0.50	-0.50
	**1-FFT**			0.79		-0.95***	-0.68		0.50	-0.99***	
	**FEH**		-0.88**				-0.73	-0.66	-0.59	-0.60	-0.87**
	**Fructo-poly**	0.83*	0.56	0.89**		-0.79	0.84*	0.80*	0.57	-0.71	0.60
	**Fructo-oligo**		-0.94**	-0.57		0.74	-0.63			0.80*	-0.88**
	**Oligo:poly ratio**	-0.74	-0.85*	-0.81*		0.70	-0.67	-0.82*	-0.74	0.63	-0.88 **
	**Reducing sugar**		-0.91**		0.62		-0.64	-0,71		-0.55	-0.82*

		WC soil		Ψwaerial		Ψwrz		*A*		WUE	
		**380**	**760**	**380**	**760**	**380**	**760**	**380**	**760**	**380**	**760**
75% plants	**A**		0.95**	0.82*	0.96**	-0,78					
	**E**					-0.86*		0.78	0.53	-0.69	-0.54
	**gs**	-0.86*	-0.98***					-0.71			
	**WUE**	0.73	0.68				0.77				
	**Aerial Biomass**					-0.59	-0.79				-0.72
	**WCaerial**			0.87*		-0.59	-0.88**	0.96**			-0.82*
	**RZ Biomass**					-0.64	-0.56				-0.75
	**WCrhizophore**	0.85*				0.57			-0.59	0.71	
	**1-SST**								0.57		
	**1-FFT**		0.82*		0.87*				0.71	0.98***	0.77
	**FEH**	0.59	0.96**		0.89**		0.82*		0.85*	0.73	0.79
	**Fructo-poly**		0.84*	0.68	0.79	-0.51	0.56	0.85**	0.91**	-0.61	
	**Fructo-oligo**	-0.83*		-0.84*		0.51	-0.67	0.73		-0.51	-0.58
	**Oligo:poly ratio**		-0.72	-0.81*	-0.56	0.78	-0.95	-1.00***	-0.61		-0.61
	**Reducing sugar**		-0.71	-0.69	-0.62		-0.60	-0.78	-0.78	0.60	

		WC soil		Ψwaerial		Ψwrz		*A*		WUE	
		**380**	**760**	**380**	**760**	**380**	**760**	**380**	**760**	**380**	**760**
50% plants	**A**	0.97***	0.76	0.93**	0.97***						
	**E**				0.63	-0.90**	-0.63	0.60	0.59		-0.53
	**gs**	-0.87*	-0.93**	0.68			-0.56	-0.95**	0.65		
	**WUE**					0.71	0.83*	0.55			
	**Aerial Biomass**					-0.95**	-0.56				
	**WCaerial**		-0.71			0.89**	-0.78			0.81*	
	**RZ Biomass**					0.51					0.67
	**WCrhizophore**	0.89**	0.62	0.76				0.74		0.88**	
	**1-SST**				-0.60	-0.53	0.69	0.64			0.81*
	**1-FFT**	0.94**	0.90**	0.75	0.62		0.70	0.89**	0.58	0.86*	
	**FEH**	0.82*		0.57		0.56	0.71	0.70		0.98***	0.97***
	**Fructo-poly**			0.56							0.73
	**Fructo-oligo**						-0.71				
	**Oligo:poly ratio**	-0.80*	-0.60	-0.57		-0.62	-0.77	-0.65		-0.99***	-0.96**
	**Reducing sugar**		-0.62			-0.77				-0.54	

		WC soil		Ψwaerial		Ψwrz		*A*		WUE	
		**380**	**760**	**380**	**760**	**380**	**760**	**380**	**760**	**380**	**760**
25% plants	**A**	0.90**	0.98***	0.84*	0.74	0.56	0.99***				
	**E**	0.94**	0.76		1.00***		0.89**	0.84*	0.82*		
	**gs**	0.97***	0.98***	0.86*	0.81*		0.98***	0.97***	0.99***		
	**WUE**			0.52	-0.84*	0.64					
	**Aerial Biomass**		-0.64	0.53		0.59				1.00 ***	
	**WCaerial**	0.70		1.00 ***	0.66			0.53			-0.86*
	**RZ Biomass**		-0.82*		-0.60		-0.61		-0.69	0.85*	
	**WCrhizophore**		0.81*		0.98***	0.96**	0.89**	0.70	0.84*		
	**1-SST**			0.78	-0.76	0.83*		0.70		0.92**	0.65
	**1-FFT**	0.91**	0.92**	0.85*	0.85*	0.53	0.87*	1.00 ***	0.88**		
	**FEH**	0.87		0.66	-0.59	0.63		0.98 ***			
	**Fructo-poly**		-0.80	0.80	-0.99 ***	-0.63	-0.91**		-0.86*		
	**Fructo-oligo**	-0.78	-0.84			-0.54	-0.81	-0.81	-0.86*		-0.59
	**Oligo:poly ratio**	-0.86*		-0.96**				-0.70			-0.84
	**Reducing sugar**		-0.76		-0.97***	-0.97***	-0.93**	-0.52	-0.87*		

WC soil: soil water content; Ψwaerial : aerial water potential; Ψwaerial: rhizophore water potential; *A*: net assimilation; *E*: transpiration; gs: stomatal conductance; WUE: water use efficiency; WCaerial: aerial water content; RZ Biomass: rhizophore biomass; WCrhizophore: rhizophore water content; SST: sucrose: sucrose-fructosyltransferase; FFT: fructose: fructose fructosyltransferase; FEH: frutano exohydrolase; fructo-poly: fructo-polysaccharide; fructo-oligo: fructo-oligosaccharide; oligo:poly ratio: fructo-oligosaccharide: fructo-polysaccharide ratio. * P<0,05; ** P<0,01; *** P<0,001.
